# Detection of long non–coding RNA homology, a comparative study on alignment and alignment–free metrics

**DOI:** 10.1186/s12859-018-2441-6

**Published:** 2018-11-06

**Authors:** Teresa M. R. Noviello, Antonella Di Liddo, Giovanna M. Ventola, Antonietta Spagnuolo, Salvatore D’Aniello, Michele Ceccarelli, Luigi Cerulo

**Affiliations:** 1 Dep. of Science and Technology, University of Sannio, via Port’Arsa, 11, Benevento, 82100 Italy; 2 BioGeM, Institute of Genetic Research “Gaetano Salvatore”, Camporeale, Ariano Irpino (AV), 83031 Italy; 3 Buchmann Institute for Molecular Life Sciences, Goethe University, Max-von-Laue-Straße 13, Frankfurt am Main, 60438 Germany; 4 Genomix4Life S.r.l., Via Salvador Allende, Baronissi (SA), 84081 Italy; 5 Dep. of Biology and Evolution of Marine Organisms, Stazione Zoologica “A. Dohrn”, Villa Comunale, Napoli, 80121 Italy

**Keywords:** Long ncRNA, Homology, String similarity

## Abstract

**Background:**

Long non-coding RNAs (lncRNAs) represent a novel class of non-coding RNAs having a crucial role in many biological processes. The identification of long non-coding homologs among different species is essential to investigate such roles in model organisms as homologous genes tend to retain similar molecular and biological functions. Alignment–based metrics are able to effectively capture the conservation of transcribed coding sequences and then the homology of protein coding genes. However, unlike protein coding genes the poor sequence conservation of long non-coding genes makes the identification of their homologs a challenging task.

**Results:**

In this study we compare alignment–based and alignment–free string similarity metrics and look at promoter regions as a possible source of conserved information. We show that promoter regions encode relevant information for the conservation of long non-coding genes across species and that such information is better captured by alignment–free metrics. We perform a genome wide test of this hypothesis in human, mouse, and zebrafish.

**Conclusions:**

The obtained results persuaded us to postulate the new hypothesis that, unlike protein coding genes, long non-coding genes tend to preserve their regulatory machinery rather than their transcribed sequence. All datasets, scripts, and the prediction tools adopted in this study are available at https://github.com/bioinformatics-sannio/lncrna-homologs.

**Electronic supplementary material:**

The online version of this article (10.1186/s12859-018-2441-6) contains supplementary material, which is available to authorized users.

## Background

Recent advances in high-throughput sequencing have led to the discovery of a substantial transcriptome portion, across different species, that does not show encoding potential [1]. Long non-coding RNAs (lncRNAs) have emerged as important players in different biological processes, from development and differentiation to multilevel regulation and tumor progression [2]. The rapidly increasing number of evidence relating lncRNAs to important biological roles and diseases [3, 4] increased the interest in developing advanced computational approaches for their identification and annotation [5–7]. However, despite their abundance and importance, their evolutionary history still remain unclear. As observed in many studies, the sequence conservation of lncRNAs is lower than protein coding genes, especially among distant species, and higher when compared to random or intronic sequences [8–10].

It has also been argued that conservation should be more preserved on RNA secondary structure functional sites than on nucleotide sequences [11]. However, as claimed recently by Rivas et al. [12], in several cases no evidence for selection on preservation of specific secondary structure has been reported till now. Conversely, promoter regions of lncRNAs appear to be generally more conserved than protein-coding genome counterparts, especially in mammalian species [1, 13]. In addition, lncRNA promoters show the presence of common binding sites for known transcription factors [14, 15], indicating that although the genomic sequences might not be highly conserved, their transcriptional machinery could be. These findings underpin the opportunity to investigate for a sequence similarity measure that is able to capture such kind of conservation, especially in promoter regions, and is computationally efficient for the detection of lncRNA homologs at genomic scale level among different species.

Current homology detection approaches, mainly based on alignment algorithms like Blast, assume the equivalence between homology and nucleotide sequence similarity. Among them, BlastR, a method that uses di-nucleotide conservation in association with BlastP to discover distantly related protein coding homologs [16], has been applied also for lncRNA homology prediction between human and other mammals [17, 18]. Approaches based on Blast–like algorithms are also the basis of lncRNA homology databases pipelines, such as NONCODE^1^ and ZFLNC^2^. However, such sets of homologs certainly represent a fraction of the whole set of conserved functions because Blast–like algorithms are designed subsuming the evolution model of proteins that could not work for lncRNAs. So, new algorithms able to capture lncRNA conservation patterns are demanded to solve this gap.

In this study, we investigate whether other kind of sequence similarity metrics, not necessarily based on sequence alignment, can achieve such a task. Our investigation spans from alignment–based metrics, widely used for searching protein coding homologs, to a representative sample of alignment–free metrics, based on information theory, frequency analysis, and data compression. Specifically we consider two alignment–based metrics, Smith–Waterman (SW) and Damerau–Levenshtein (DLevDist) distance (Table 1); and 8 alignment-free metrics (Table 2), including: *n*-gram distance (qgram), Cosine similarity (cosine), Jaccard similarity (jaccard), Base–Base Correlation distance (BBC), Average Common Substring distance (ACS), Lempel–Ziv complexity distance (LZ), Jensen–Shannon distance (JSD), and Hamming distance (HDist). Alignment–free metrics have been chosen by their popularity in other disciplines and because in our knowledge have never been adopted for homology identification.

**Table 1:** Definition of the adopted homology metrics (Alignment–based)

Metric	Definition	Description
Smith–Waterman similarity	$SW(X,Y) = \max \limits _{\substack {\scriptscriptstyle x \in seq(X)\\ \scriptscriptstyle y \in seq(Y)}} \left (\frac {sw(x,y)}{len(x)+len(y)}\right)$	The Smith–Waterman similarity *sw*(*x,y*) is given by maximizing a score computed over a number of operations needed to transform one string into the other, where an operation is defined as an insertion, deletion, or substitution of a single character [46]. Deletions/insertions (gaps) are penalized with a zero score, matches are rewarded with +5, and substitutions are penalized with -4 (NUC 4.4 substitution matrix). The time complexity is *O*(*len*(*x*)·*len*(*y*)).
Damerau–Levenshtein distance	$DLevDist(X,Y) = \min \limits _{\substack {\scriptscriptstyle x \in seq(X)\\ \scriptscriptstyle y \in seq(Y)}} \left (\frac {dl(x,y)}{len(x)+len(y)}\right)$	The Damerau–Levenshtein distance *dl*(*x,y*) is given by counting the minimum number of operations needed to transform one string into the other, where an operation is defined as an insertion, deletion, or substitution of a single character, or a transposition of two adjacent characters [47]. The time complexity is *O*(*len*(*x*)·*len*(*y*)).

*X* and *Y* are two candidate long non coding genes, *seq*(*X*) and *seq*(*Y*) are the sets of representative sequences of *X* and *Y* respectively (promoter or transcript), *len*(*x*) and *len*(*y*) are the lengths of sequences *x* and *y* respectively. Where applicable a metric is normalized with respect to the sum of sequence length [42] and is minimized (maximized) for distance (similarity) metrics among all couple of transcript sequences *x*∈*seq*(*X*),*y*∈*seq*(*Y*)

**Table 2:** Definition of the adopted homology metrics (Alignment–free)

Metric	Definition	Description
n-gram distance	${qgram}_{n}(X,Y) = \min \limits _{\substack { \scriptscriptstyle x \in seq(X)\\ \scriptscriptstyle y \in seq(Y)}} \left (\frac {\sum _{i}|q^{x}_{i}-q^{y}_{i}|}{len(x)+len(y)}\right)$	A *n*-gram is a subsequence of *n* consecutive characters of a string [48]. If $\mathbf {q}^{x} = \left (q^{x}_{1}, q^{x}_{2}, \dots, q^{x}_{K}\right)$ is the *n*-gram vector of counts of *n*-gram occurrences in the sequence *x* the *n*-gram distance is given by the sum over the absolute differences $|q^{x}_{i}-q^{y}_{i}|$, where $q^{x}_{i}$ and $q^{y}_{i}$ are the i-th unique *n*-grams of *x* and *y* respectively obtained by sliding a window of *n* characters wide over *x* and *y* and registering the occurring *n*-grams. The time complexity is *O*(*len*(*x*)·*len*(*y*)).
Cosine similarity	${cosine}_{n}(X,Y) = \max \limits _{\substack {\scriptscriptstyle x \in seq(X)\\ \scriptscriptstyle y \in seq(Y)}} \frac {\mathbf {q}^{x} \cdot \mathbf {q}^{y}}{\|\mathbf {q}^{x}\|\|\mathbf {q}^{y}\|}$	The cosine similarity is the cosine of the angle between the two *n*-gram vectors **q**^*x*^ and **q**^*y*^ [40]. The time complexity is *O*(*len*(*x*)+*len*(*y*)).
Jaccard similarity	${jaccard}_{n}(X,Y) = \max \limits _{\substack {\scriptscriptstyle x \in seq(X)\\ \scriptscriptstyle y \in seq(Y)}} \left (\frac {\sum _{i} \left (\mathbbm {1}_{q^{x}_{i}>0} + \mathbbm {1}_{q^{y}_{i}>0}\right)}{\sum _{i} \mathbbm {1}_{q^{x}_{i}>0} \cdot \mathbbm {1}_{q^{y}_{i}>0}} - 1\right)$	The Jaccard coefficient measures the similarity between two finite sets, and is defined as the size of the intersection divided by the size of the union of the sample sets [49]. The size is computed from the set of unique *n*-grams by means of $\mathbbm {1}_{q^{x}_{i} > 0}$, the indicator function having the value 1 if the i-th *n*-gram is present in *x*, 0 otherwise. The time complexity is *O*(*len*(*x*)+*len*(*y*)).
Base–base correlation distance	$BBC(X,Y) = \min \limits _{\substack {\scriptscriptstyle x \in seq(X)\\ \scriptscriptstyle y \in seq(Y)}}\sqrt {\sum _{i=1}^{16}(V_{x_{i}} - V_{y_{i}})^{2}}$	The Base–base correlation measures the sequence similarity by computing the euclidean distance between two 16-dimensional feature vectors, *V*_*x*_ and *V*_*y*_, which contain all base pair mutual information [50]. The time complexity is *O*(*len*(*x*)·*len*(*y*)).
Average common substring distance	$ACS(X,Y) = \min \limits _{\substack {\scriptscriptstyle x \in seq(X)\\ \scriptscriptstyle y \in seq(Y)}}\frac {1}{2} \left (\sum _{i=1}^{len(x)} \frac {lcs(x(i),y)}{len(x)} + \sum _{i=1}^{len(y)} \frac {lcs(y(i),x)}{len(y)}\right)$	The average common substring is the average lengths of maximum common substrings for constructing phylogenetic trees [51]. Specifically, the *lcs*(*x*(*i*),*y*) (*lcs*(*y*(*i*),*x*)) is the length of the longest common substring of *x* (*y*) starting at each position *i* of *x* (*y*) and exactly matching some substring in *y* (*x*). The time complexity is *O*(*len*(*x*)+*len*(*y*)).
Lempel–Ziv complexity distance	$LZ(X,Y) = \min \limits _{\substack {\scriptscriptstyle x \in seq(X)\\ \scriptscriptstyle y \in seq(Y)}}\frac {c(x,y)-c(x)+c(yx)-c(y)}{\frac {1}{2}[c(xy)+c(yx)]} $	The Lempel–Ziv complexity distance is defined by considering the minimum number of components over all production histories of *x* and *y*, *c*(*x*) and *c*(*y*) and their concatenations, *c*(*xy*) and *c*(*yx*) [52]. The time complexity is *O*(*len*(*x*)·*len*(*y*)).
Jensen–Shannon distance	$JSD(X,Y) = \min \limits _{\substack {\scriptscriptstyle x \in seq(X)\\ \scriptscriptstyle y \in seq(Y)}}\frac {1}{2}KL(V_{x},V_{M}) + \frac {1}{2}KL(V_{y},V_{M})$	The Jensen–Shannon distance is computed by averaging the Kullback–Leibler Divergence (*KL*) of *V*_*x*_ with respect to *V*_*M*_ and *V*_*y*_ with respect to *V*_*M*_, where *V*_*x*_ and *V*_*y*_ are the same 16-dimensional feature vectors defined for BBC, and $V_{M} = \frac {V_{x}+V_{y}}{2}$ [41]. The time complexity is *O*(*len*(*x*)+*len*(*y*)).
Hamming distance	$HDist(X,Y) = \min \limits _{\substack {\scriptscriptstyle x \in seq(X)\\ \scriptscriptstyle y \in seq(Y)}}hd(r(x),r(y))$	The Hamming distance is defined between two strings of the same length as the number of positions in which corresponding values are different. We adopt two bit strings of length *n*, namely *r*(*x*) and *r*(*y*), representing the regulatory transcriptional machinery of *x* and *y* respectively, and *n* is the number of all transcription factors available in JASPAR [24]. Each position *i* of such bit strings is equal to 1 if the *i*-th transcription factor binds the promoter while 0 otherwise. The time complexity is *O*(*n*).

*X* and *Y* are two candidate long non coding genes, *seq*(*X*) and *seq*(*Y*) are the sets of representative sequences of *X* and *Y* respectively (promoter or transcript), *len*(*x*) and *len*(*y*) are the lengths of sequences *x* and *y* respectively. Where applicable a metric is normalized with respect to the sum of sequence length [42] and is minimized (maximized) for distance (similarity) metrics among all couple of transcript sequences *x*∈*seq*(*X*),*y*∈*seq*(*Y*)

We evaluate the metrics in three different species, human (hg38), mouse (mm10), and zebrafish (danRer10), against a manually curated gold–standard, originated from experimentally validated lncRNA homologs collected from the literature with the support of public lncRNA databases, such as lncRNAdb [19], LNCipedia [20, 21], and lncRNome [22]. We show that some alignment–free metrics provide a better alternative to pairwise-alignment metrics, such as Smith–Waterman, especially between phylogenetically distant species. Surprisingly, in contrast with protein coding genes, lncRNA homologs exhibit higher alignment–free scores in promoter regions corroborating the hypothesis that lncRNA genes tend to preserve their regulatory machinery rather than their transcribed sequence.

## Results

Given two species *S*_1_ and *S*_2_, Tables 1 and 2 report the set of metrics, we analyze, to detect whether two genes *X*∈*S*_1_ and *Y*∈*S*_2_ are homologs or not. For discussion purposes we consider three main factors that, as expected, could affect homology prediction: i) phylogenetic distance (close or distant), assuming human–mouse as close species, while mouse–zebrafish and human–zebrafish as distant species; ii) kind of transcript (protein coding or long non-coding); and iii) sequence region (promoter or transcript). In the following we report the results obtained with three empirical experiments aimed at evaluating the effectivenes of the proposed metrics: i) evaluation against a manually curated gold–standard originated from experimentally validated lncRNA homologs (Additional file 4: Table S1), ii) evaluation agaist NONCODE and ZFLNC public annotation databases providing lncRNA homologous associations among different species detected with a Blast like pipeline, and iii) evaluation of functional concordance that looks at protein coding genes localized in the proximity of lncRNAs and measures their Gene Ontology term enrichment.

### Metrics evaluation on manually curated gold-standard

**Fig. 1:**
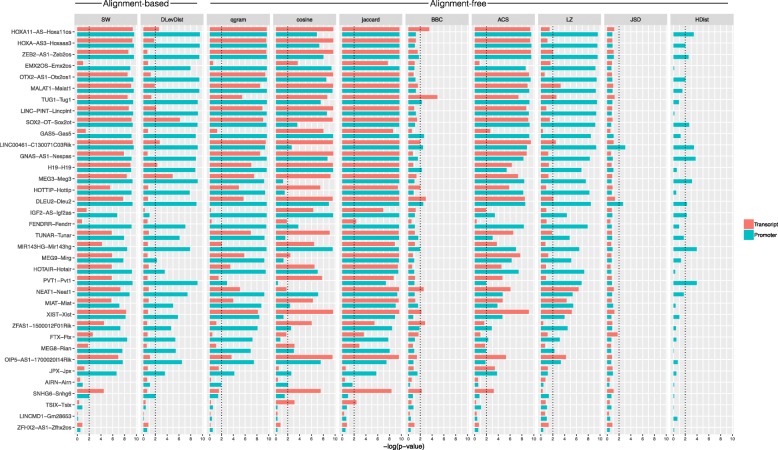
*P*-value barplot for permutation test in Human-Mouse. -log10(p-values) estimated by permutation test over a null distribution of random non–homologous pairs in Human-Mouse on promoter (blue bars) and transcript sequences (red bars) for each considered metric. Homologous lncRNA couples are ranked according to the best prediction computed on promoter sequences among metrics. The x-axis reports true homologous pairs for the two species

**Fig. 2:**
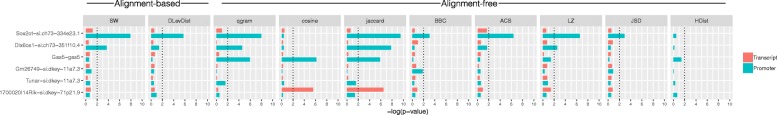
*P*-value barplot for permutation test in Mouse-Zebrafish. -log10(p-values) estimated by permutation test over a null distribution of random non–homologous pairs in Mouse-Zebrafish on promoter (blue bars) and transcript sequences (red bars) for each considered metric. Homologous lncRNA couples are ranked according to the best prediction computed on promoter sequences among metrics. The x-axis reports true homologous pairs for the two species

**Fig. 3:**
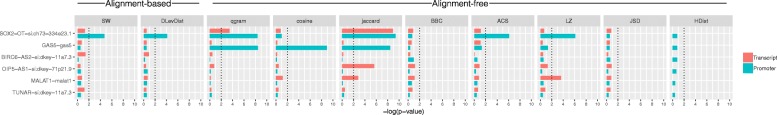
*P*-value barplot for permutation test in Human-Zebrafish. -log10(p-values) estimated by permutation test over a null distribution of random non–homologous pairs in Human-Zebrafish on promoter (blue bars) and transcript sequences (red bars) for each considered metric. Homologous lncRNA couples are ranked according to the best prediction computed on promoter sequences among metrics. The x-axis reports true homologous pairs for the two species

Figures 1, 2 and 3 show, respectively for human–mouse, mouse–zebrafish, and human–zebrafish, the −*log*(*pvalue*) for each considered metric (Tables 1 and 2) estimated by permutation test over a null distribution of non–homologous pairs randomly selected. The aim is to estimate to which extend a candidate metric is able to separate the true homologous pair from a huge set of random selected non-homologous pairs (permutation test). The set of non-homologous pairs are constructed by fixing a lncRNA candidate in a species and selecting a random set of sequences, approximately of the same length, in the other species known to be not homologous. Metrics depending on parameters were customized accordingly to obtain the best possible results. Specifically, for SW, we estimated the best levels of match gain and gap/missmatch penalty with a grid searching procedure and for HDist, we adopted the MEME FIMO tool [23] with JASPAR positional frequency matrices (PFMs) [24]. The set of non-homologous pairs is ranked according to the best prediction computed on promoter sequences among metrics.

In closer related species (human–mouse), no distinction can be observed between alignment–based and alignment–free metrics. Figure 1 shows more than 23 out of 36 true homologous pairs with a *p*-value ≤0.01 in both alignment–based and almost all alignment–free metrics. Conversely, alignment–free metrics, especially jaccard and qgram, are more suitable among phylogenetically distant species. Jaccard exhibits a *p*-value ≤0.01 in 3 out of 6 true homologous pairs (Figs. 2 and 3). Instead, some metrics, such as DLevDist, BBC and JSD, are less powerful to detect homologous lncRNAs.

Moreover some couples failed to be detected regardless to the used metrics or sequence region. For example, for ZFHX2-AS1–Zfhx2os (Fig. 1) the literatrure suggests that a conservation of transcriptional profiles could be observed and that only a small genomic region, which perhaps contains important signals for the antisense transcription, could be considered conserved between human and mouse [25]. Similarly, the conservation of TUNAR involves only a small transcript region (about the 8% of the entire human sequence) that interacts with several RNA–binding proteins (as PTBP1 and hnRNP-K) responsible of functional conservation in all the considered species [26].

The sequence region (transcript vs. promoter) seems to play an important role only in phylogenetically distant species, with the exception of few cases. In Fig. 1 the number of significant true homologous pairs detected by each metric is higher for promoters in 5 cases out of 10 in human-zebrafish (Fig. 2), while such cases are 8 out of 10 in mouse-zebrafish (Fig. 3).

In phylogenetically close species (human–mouse), only few cases are affected by sequence region. For example, promoter sequence seems to be crucial for the functional maintenance of JPX (XIST Activator) in mammal species, differently from TSIX (XIST Antisense RNA), where the transcript provides uniquely the information of conservation. According to the corresponding literature, the promoter of JPX has been shown to interact with the Xist promoter in undifferentiated embryonic stem cells [27], while TSIX seems to be involved in the modulation of chromatin modification status of Xist promoter, suggesting a conserved function in mammals carried by the transcript structure [28].

In distant species, alignment–based metrics are able to detect a lower number of homologous lncRNAs. This is probably related to the regulatory machinery that alignment–based metrics are less prone to detect.

### Consensus with NONCODE and ZFLNC pipelines

**Fig. 4:**
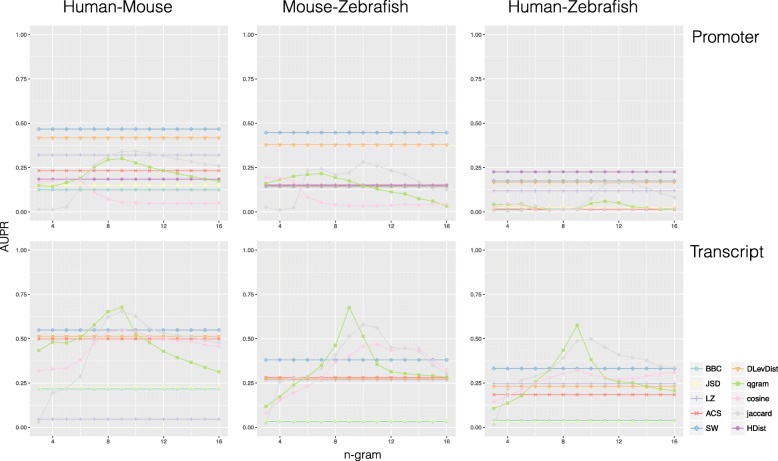
NONCODE AUPR plots. Metric prediction performance computed on promoter and transcript sequences for NONCODE lncRNA homologs (AUPR on y-axis and *n*, the number of consecutive nucleotides in *n*-gram metrics, on x-axis)

**Fig. 5:**
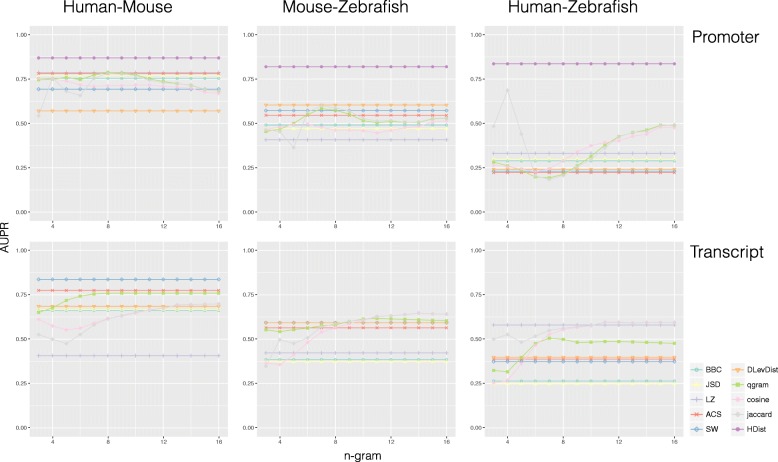
ZFLNC AUPR plots. Metric prediction performance computed on promoter and transcript sequences for ZFLNC lncRNA homologs (AUPR on y-axis and *n*, the number of consecutive nucleotides in *n*-gram metrics, on x-axis)

Figures 4 and 5 show the prediction performances, in terms of AUPR (Area under the Precision–Recall curve) plots, obtained by each metric with two database annotations, respectively NONCODE and ZFLNC. The x-axis reports the number *n* of consecutive characters considered for gram–based metrics. This means that remaining metrics are shown as horizontal lines since they do not depend on *n*. As baseline comparison, we computed AUPR also for a random set of protein coding genes (Additional file 1: Figure S1). Additional files 2: Figure S2 and 3: Figure S3 show also the ROC curves obtained respectively in NONCODE and ZFLNC.

SW, jaccard and cosine with *n* greater than 10 perform well when applied to protein coding transcript sequences, confirming that those metrics, in particular SW, are suitable for identifying homologous coding gene in both phylogenetically close and distant species. An opposite behaviour can be observed when comparing promoter sequences. In both phylogenetically close and distant species, the similarity of promoter regions seems to predict better the homology of lncRNAs rather than protein coding genes. In particular, HDist results to be the best predictor in ZFLNC (Fig. 2), reflecting the evidences regarding regulatory programs [29] and conservation status [1, 30] of lncRNAs with respect to protein coding genes. Furthermore, according to the manually curated gold-standard results, some metrics, such as BBC, JSD and LZ, seem to be not suitable for the detection of homology, both in protein coding genes and in lncRNAs (AUPR less than 0.5 in mouse–zebrafish and less than 0.4 in human–zebrafish).

The conservation degree of lncRNA homologs is mainly affected by evolution distance, reflecting the evidences, shown also in the manually curated gold-standard, that lncRNAs evolve more rapidly. It is possible to observe that AUPR decreases with the increase of species distance for almost all metrics. For example, the AUPR of SW in NONCODE decreases from a 0.55 in human–mouse to 0.45 in mouse–zebrafish and to 0.33 in human–zebrafish (Fig. 1). While, the AUPR of jaccard and cosine in ZFLNC decrease from a 0.78 and 0.77 in human–mouse to 0.64 and 0.61 in mouse–zebrafish and to 0.59 and 0.50 in human–zebrafish, respectively.

Although semi–automatic generated gold-standards present major biases related to underlying automatic pipelines based on BLAST, some of conclusions, drawn with the manually curated gold-standard, are still supported, making the empirical evidence reinforced by a more representative statistical population.

### Genome functional concordance analysis

**Fig. 6:**
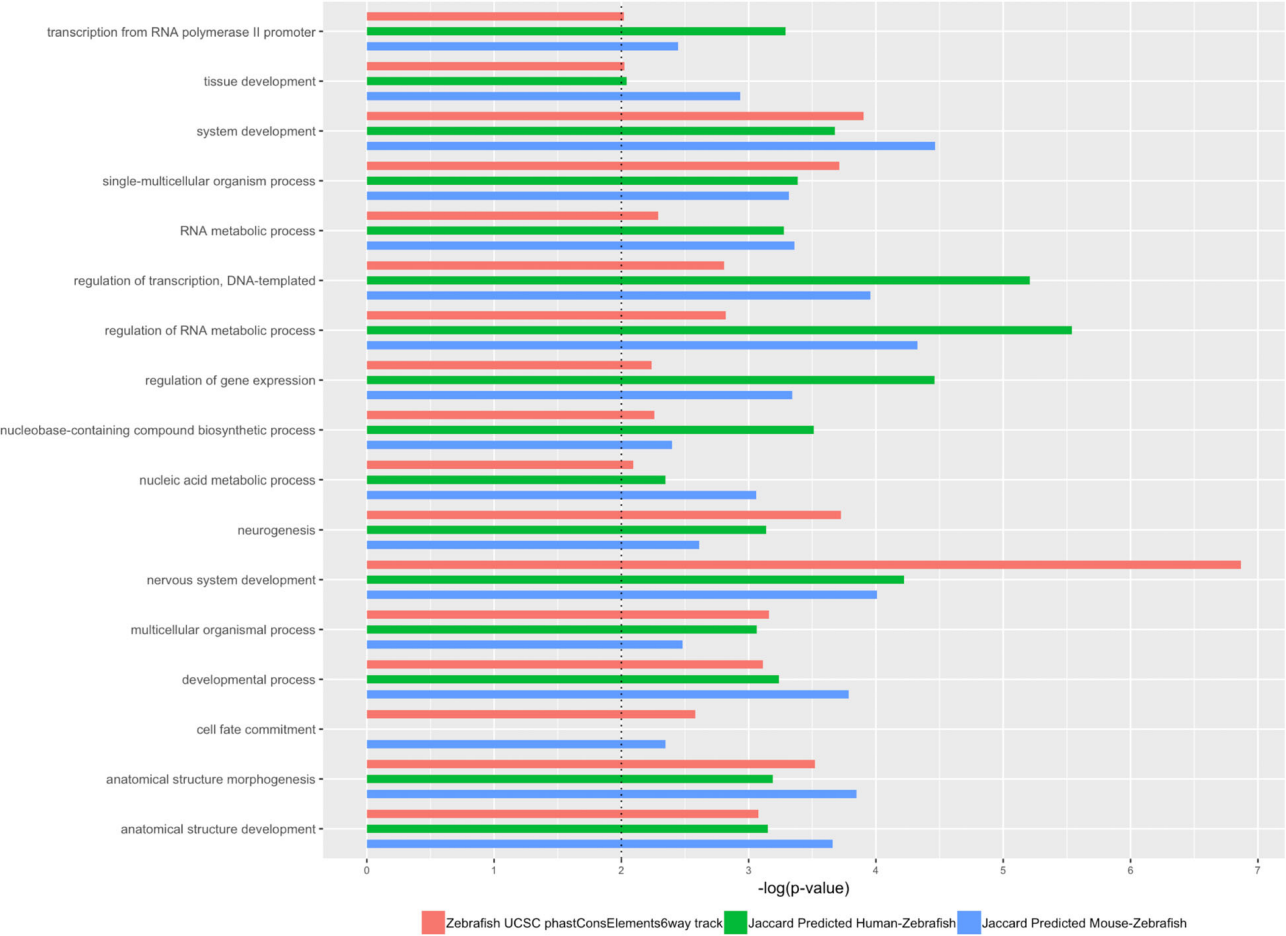
Functional concordance plots. GO Biological Process (BP) terms enrichment of flanking protein coding genes of lncRNAs overlapping the conserved elements in Zebrafish (green bars) and predicted to be homologs according to Jaccard similarity with *n*=12 (red bars) in Human and Mouse. Blue bars indicate the percentages from the entire transcriptome of the specific specie of the BP terms

**Fig. 7:**
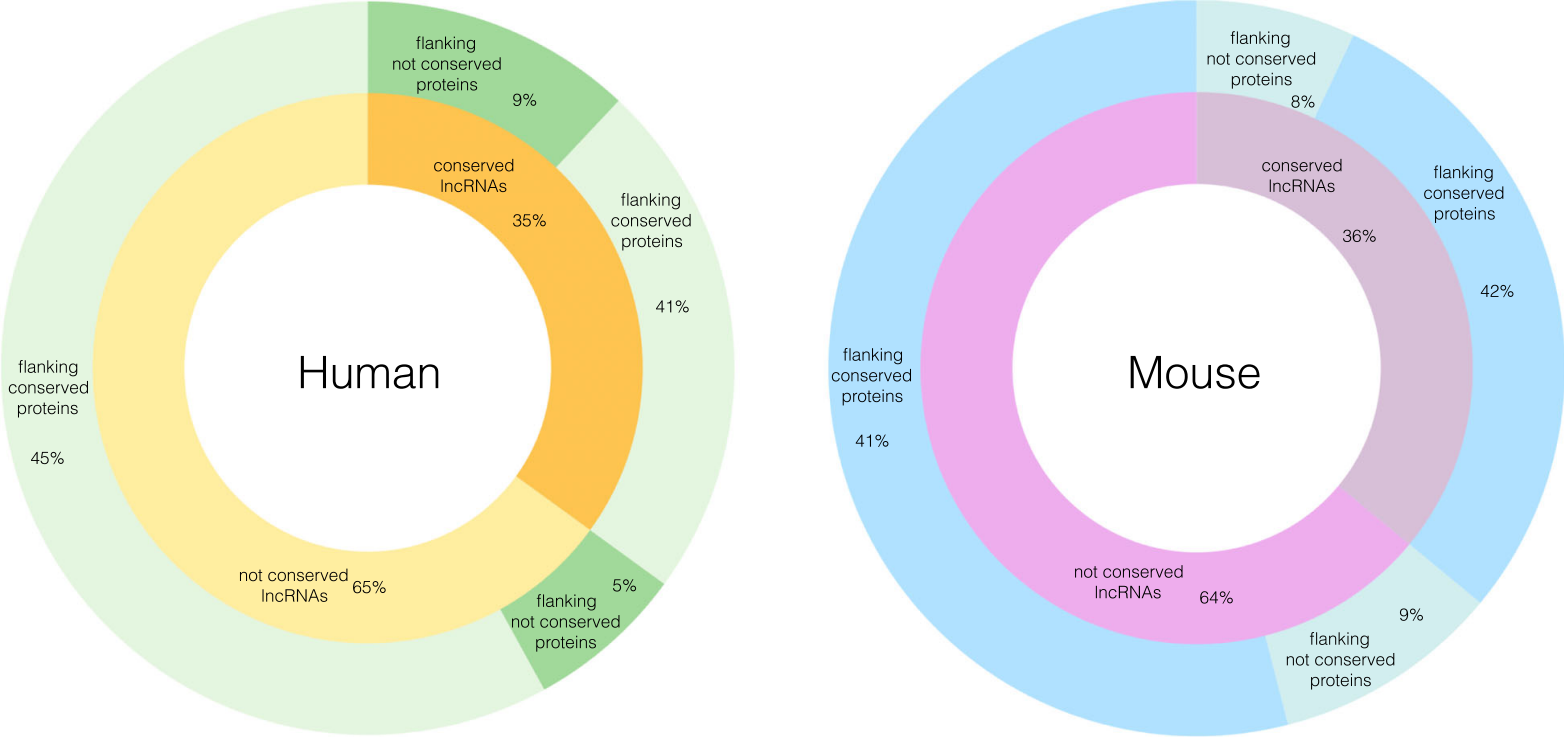
Distribution of conserved and non conserved flanking genes

In order to assess the ability of alignment–free metrics to predict conservation of lncRNAs also regarding to their known and preserved biological functionality, we performed a GO enrichment analysis considering the nearest protein coding genes flanking the sets of zebrafish lncRNAs predicted to be orthologs in human and mouse (using jaccard with *n*=12). We adopted jaccard similarity as predictor since this metric in the previous empirical analyses showed in average a good prediction performance, but similar results can be obtained also with other alignment–free metrics (data not shown). As baseline, we considered the protein coding genes flanking the lncRNAs that overlap the most significantly conserved elements produced by the phastCons program [31] from zebrafish genome. Significantly enriched GO Biological Process (BP) terms (*p*-value ≤0.01) were obtained using DAVID functional annotation tool [32] and redundant enriched GO terms were removed using Revigo [33] (Additional file 5: Table S2). For each enriched GO category, the percentages of genes overlapping the most significantly conserved elements are also shown. Figure 6 shows the grouped BP terms that resulted to be enriched in all three considered sets: the jaccard predicted zebrafish lncRNA orthologs in human and mouse, and the phastCons conserved lncRNAs. As expected and in according to several studies describing lncRNA functional roles shared by different species [34–37], the enriched categories include development at several stages, regulation of transcription, and metabolic processes. On average, it can be observed an increment in terms of enrichment of the ultra–conserved GO terms considering the sets of zebrafish lncRNAs predicted to be orthologs in human and mouse. However, it is not surprising that in few cases the GO term enrichment related to the ultra–conserved set is higher that the ones predicted using jaccard similarity. For example, it is known that lncRNAs play critical roles in the development of nervous system (neurogenesis) and that approximately 40% of lncRNAs are expressed in the brain in a tissue specific manner[17]. Moreover, these brain–specific lncRNAs show the highest signals of evolutionary conservation in comparison with those expressed in other tissues [38]. Figure 7 shows the percentages of predicted zebrafish lncRNA orthologs in human and mouse conserved or not with a zebrafish phastCons element and the corresponding percentages of flanking coding genes overlapping or not the same regions of conservation. The observed similarity at functional level in both species given by the GO enrichment analysis is not due to an over-representation of conserved lncRNA ortologs (35% in Human and 36% in Mouse). As expected, the high number of flanking coding genes within the zebrafish phastCons elements reflect the general feature of lncRNAs to be involved in vertebrate shared functional processes through in *cis* expression regulation of nearby conserved genes. This result constitutes a further proof that alignment-free metrics, such as Jaccard similarity, work alongside typical approaches based on pure conservation among species, and are able to identify additional orthologs not included in the typical multi–alignment conservation track.

## Discussion

In this study, we provide a systematic assessment of alignment-based and alignment-free metrics to investigate the conservation of lncRNAs looking at both promoter and transcript sequences in human, mouse and zebrafish. We evaluate the metrics against a manually curated gold-standard of validated lncRNA homologs available in literature. We show how alignment-free metrics could represent a powerful alternative to alignment metrics to detect lncRNA homology, especially in phylogenetically distant species and promoter regions. Despite the under-representation of considered gold-standard, alignment–free metrics, and in particular jaccard, could represent an optimal tradeoff between efficiency and efficacy for large scale genome annotation.

These findings are also supported by an extended empirical evaluation on two semi-automatic generated gold-standard, collected from lncRNA annotation databases as NONCODE and ZFLNC. It is important to specify that, although the necessity of retrieving an increased number of homologous lncRNA couples than that collected in the manually curated gold-standards, the semi-automatic generated gold-standard present several weaknesses, due to the massive automatic Blast based pipeline biases.

Our results reflect the rapid evolution of lncRNAs, divergent even between closely related species, confirmed by the fact that 81% of lncRNA families are only primate specific [17]. The promoter regions of lncRNA genes are generally more conserved than promoters of protein-coding genes [1] and encode crucial information that is better detected with alignment-free metrics, such as jaccard, suggesting a sustained selective pressure acting on these sequences. The evolution of transcription factor binding sites follow usually patterns marked by relocations and transpositions inside the promoter region. This preserves the regulatory machinery but limit sub-sequence similarity. Alignment–based metrics in preserving the relative order of common sub-sequences are able to detect point mutations, deletion, and insertion of small sequences but are not able to detect re-locations, crossovers, and/or transpositions as alignment–free metrics can do. Genome functional concordance analysis confirm that conservation captured at promoter level by alignment–free metrics is highly consistent with the preservation of their biological functionality between species carried by coding genomic neighbourhood. This make us to suppose that lncRNA homologs tend to preserve their regulatory relationships more than their transcribed sequence.

## Conclusions

We proposed the use of alignment–free metrics to investigate the mechanism of conservation of long non-coding RNAs in three different species. To some extent, we found that n-gram metrics, when applied to promoter regions, are able to capture lncRNA homology associations between close and distant species. The obtained results persuaded us to formulate a hypothesis of conservation schema that impacts the promoter regions of lncRNAs. This mechanism suggests that lncRNAs tend to preserve the regulatory relationship with transcription factors rather than the information encoded in their sequence. As our results are limited to the three species, human, mouse, and zebrafish, it is unquestionable that more data on different species and a larger manually curated gold-standard are crucial to generalize the mechanism of conservation governing the evolution of lncRNAs.

## Methods

### Sequence similarity metrics

Given two species *S*_1_ and *S*_2_, Tables 1 and 2 report the set of metrics, we analyze, to detect whether two genes *X*∈*S*_1_ and *Y*∈*S*_2_ are homologs or not. We consider two alignment-based metrics, Smith–Waterman similarity and Damerau–Levenshtein distance (Table 1), widely adopted to detect protein coding homology [39], and several alignment-free metrics (Table 2), including: *n*-gram and common substring based distances, adopted in text mining and information retrieval [40]; two factor frequencies distances, Base–base correlation and Jensen–Shannon Divergence test, adopted in genome comparison [41]; Lempel–Ziv complexity distance based on data compression; and Hamming distance adapted to compute the concordance between regulatory transcriptional machinery of promoter sites. To make a measure comparable among sequences with different lengths, where applicable, a metric is normalized with respect to the sum of sequence lengths [42]. A gene *X* is modeled as a set of sequences *seq*(*X*) extracted from a genome. In particular, we consider two types of sequence sets: the set of transcribed sequences and the set of promoter regions. A transcribed sequence is constructed by merging all exons belonging to that transcript, while a promoter region is built by considering the conventionally 2000 bp up and 1000 bp down stream from the transcription starting site. A metric is computed for all possible pairs of sequences belonging to the two sets representing the two candidate genes. Among all measures the minimum is considered if the metric is defined as a distance, instead the maximum if the metric is defined as a similarity.

### Metrics evaluation on manually curated gold-standard

We evaluate the metrics in three different species, human (hg38), mouse (mm10), and zebrafish (danRer10), against a manually curated gold–standard, originated from experimentally validated lncRNA homologs (Additional file 4: Table S1). It has been collected from the literature with the support of: lncRNAdb [19], a database that provides annotations of eukaryotic lncRNAs; LNCipedia [20, 21]; and lncRNome [22], a knowledge-base compendiums of human lncRNAs. Table 3 reports the number of collected lncRNA homologs between human and mouse, mouse and zebrafish, and human and zebrafish.

**Table 3:** Annotated homologous genes between species in manual curated gold-standard

Gene class	Gene class	Human	Human	Mouse
Specie1	Specie2	Mouse	Zebrafish	Zebrafish
Antisense	Antisense	12	2	1
Antisense	lincRNA	8	2	0
lincRNA	Antisense	1	1	2
lincRNA	lincRNA	20	2	2
Overlapping	Overlapping	1	1	1
Total lncRNAs		42	8	6
Protein coding	Protein coding	12998	10209	10126

Due to the limited number of collected homologous pairs, we report to which extend (*p*-value) a candidate metric is able to separate the true homologous pair from a huge set of random selected non-homologous pairs (permutation test). The set of non-homologous pairs are constructed by fixing a lncRNA candidate in a species and selecting a random set of sequences, approximately of the same length, in the other species known to be not homologous.

### Consensus with NONCODE and ZFLNC pipelines

NONCODE and ZFLNC are public annotation databases providing lncRNA homologous associations among different species. Such associations are detected by classical sequence homology pipelines based on multi alignment metrics such as those adopted to identify protein coding homologs. Specifically, NONCODE provides conservative and evolutionary status of stored lncRNAs through a genome comparison conservation analysis based on UCSC LiftOver tool; while, ZFLNC provides zebrafish lncRNA functions and homologs identified through a pipeline based on: BLASTn, collinearity with conserved coding gene, and overlap with multi-species ultra-conserved non-coding elements.

Although such databases cannot be adopted as a typical gold–standard because the sample is biased on the similarity metric used in the original discovery pipelines, we still perform an evaluation against database annotations. The aim is to show to which extend alignment–free metrics reproduces the state of art of lncRNA homologs annotated with pipelines based essentially on alignment–based metrics.

From NONCODE we selected 882 human lncRNA sequences having 44 homologous counterparts in zebrafish and 523 in mouse. From ZFLNC we selected 676 zebrafish lncRNA sequences presenting a counterpart both in human and mouse. Prediction accuracy is evaluated with area under the Precision and Recall curve (AUPR), since it gives more information when dealing with highly skewed datasets [43, 44]. Specifically, we provide a normalized version of AUPR that takes into account the unachievable region in PR space, as proposed in Kendrick et al. [44], that allows to compare performances estimated on datasets with different class skews. In additional data we provide also ROC plots.

### Genome functional concordance analysis

It is generally assumed that homologous genes play similar biological roles in different species [45]. Since Gene Ontology (GO) analysis can be considered as a good in-silico indicator of biological function, we provide an alternative assessment strategy that evaluates the functional concordance of lncRNA homologs candidates. This strategy, adopted similarly in Basu et al. [18], looks at protein coding genes localized in the proximity of lncRNAs (within a window of 1 mb) and measures their GO term enrichment in Biological Processes (BP) with DAVID tool [32].

As case study we evaluate the functional concordance on a set of lncRNA zebrafish homologous candidates predicted from a sample of 1000 random lncRNAs belonging to human and mouse. As baseline, we consider zebrafish lncRNAs belonging to ultra–conserved regions obtained with UCSC phastConsElements6way tracks. This provided us a set of enriched GO terms that can be assumed to be the most conserved biological function among the considered species [34–37]. The idea is to compare the baseline enrichment with the enrichment of predicted lncRNAs flanking protein coding genes. An increment of the latter enrichment means that predicted lncRNAs are able to capture additional flanking proteins not revealed in canonical phastConsElements6way tracks, corroborating the hypothesis that such lncRNAs, in controlling such flanking genes, should contribute to the ultra-conserved biological function.

## Additional files

Additional file 1 Additional Figure 1. Protein-coding gene AUPR plots. Metric prediction performance computed on promoter and transcript sequences for annotate protein-coding homologs (AUPR on y-axis and n, the number of consecutive nucleotides in n-gram metrics, on x-axis). (PDF 158 kb)

Additional file 2 Additional Figure 2. NONCODE ROC curves. ROC curves computed on promoter and transcript sequences for NONCODE lncRNA homologs (for n-gram metrics, *n*=12 has been chosen). (PDF 822 kb)

Additional file 3 Additional Figure 3. ZFLNC ROC curves. ROC curves computed on promoter and transcript sequences for ZFLNC lncRNA homologs (for n-gram metrics, *n*=12 has been chosen). (PDF 1580 kb)

Additional file 4 Additional Table 1. Manually curated gold–standard. Experimentally validated lncRNA homologs for the considered species. (XLSX 13 kb)

Additional file 5 Additional Table 2. GO biological process enriched terms. DAVID results for GO enrichment analysis of flanking proteins of Zebrafish lncRNA predicted to be homologous in Human (Sheet 1), Mouse (Sheet 2) and of lncRNA overlapping the conserved elements in Zebrafish (Sheet 3). (XLSX 21 kb)

## Footnotes

1
http://www.noncode.org

2
http://www.zflnc.org
