# Supplementary figures and images for: Detection of long non–coding RNA homology, a comparative study on alignment and alignment–free metrics
Source: BMC Bioinformatics. 2018 Nov 6;19:407. doi: 10.1186/s12859-018-2441-6 (PMC6220562; doi:10.1186/s12859-018-2441-6)

Human-Mouse

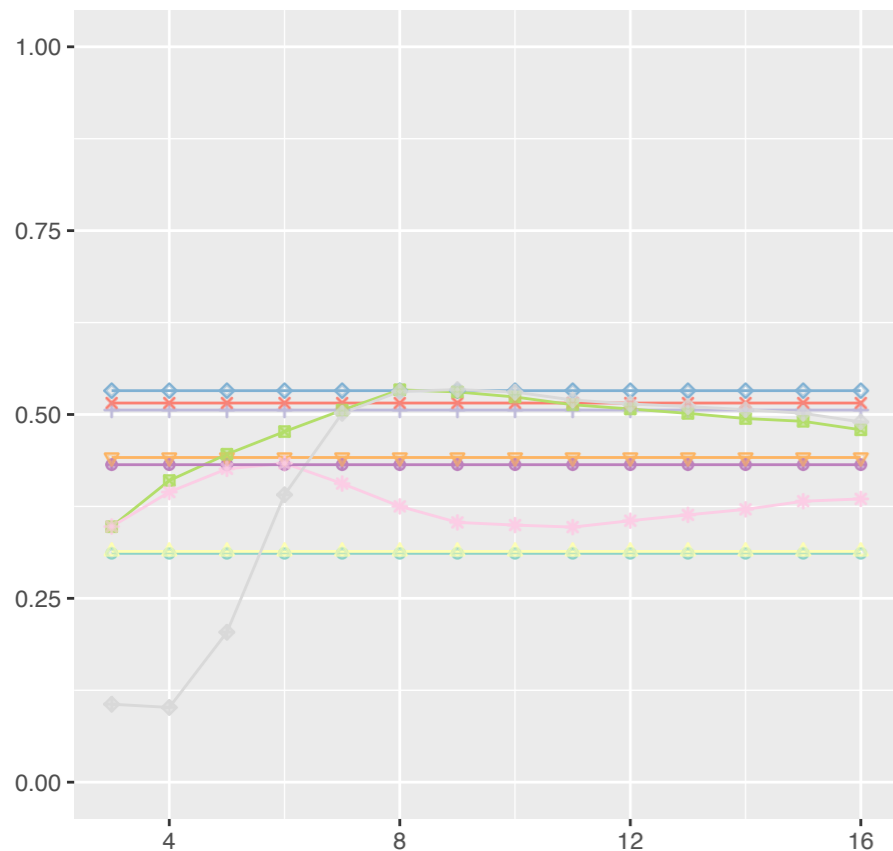

Mouse-Zebrafish

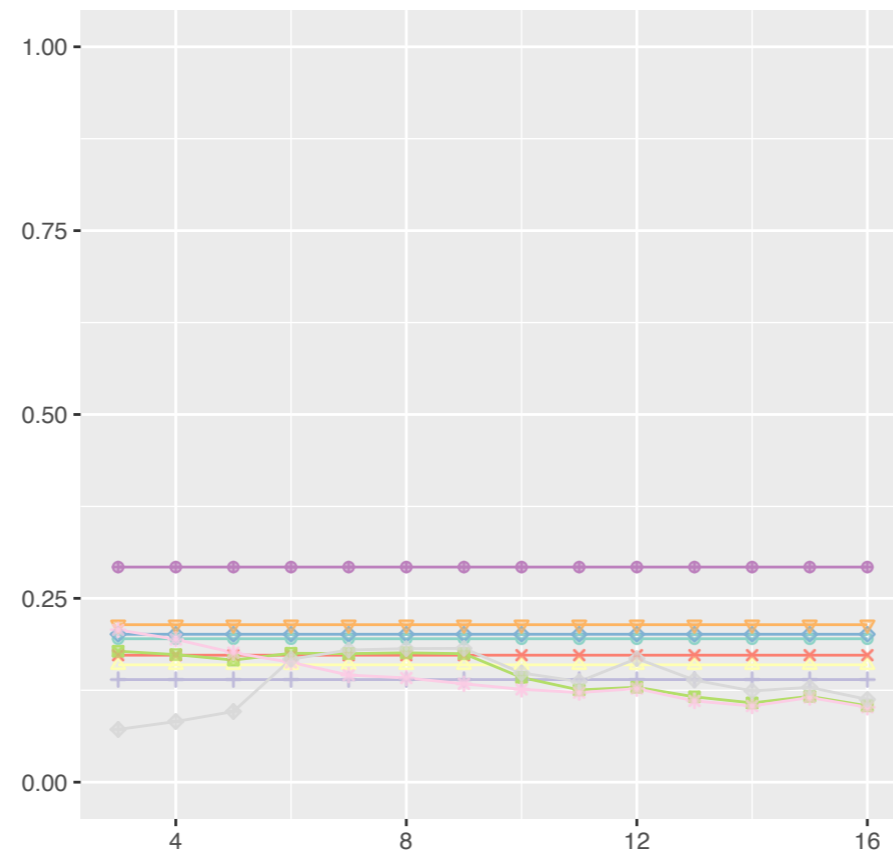

Human-Zebrafish

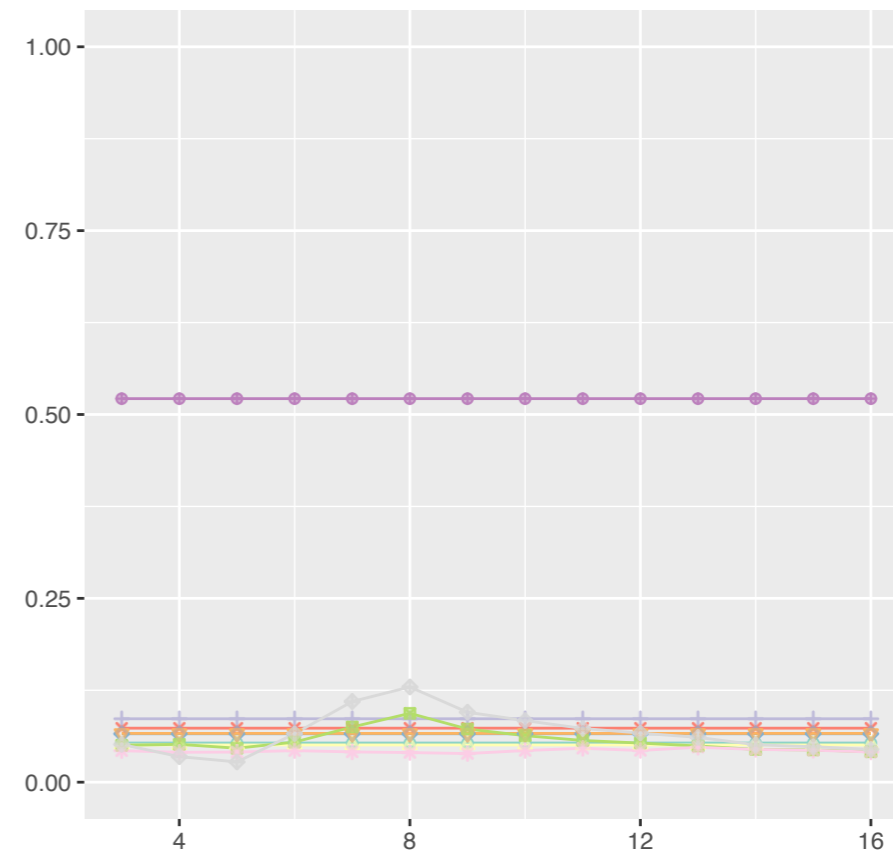

Promoter

AUPR

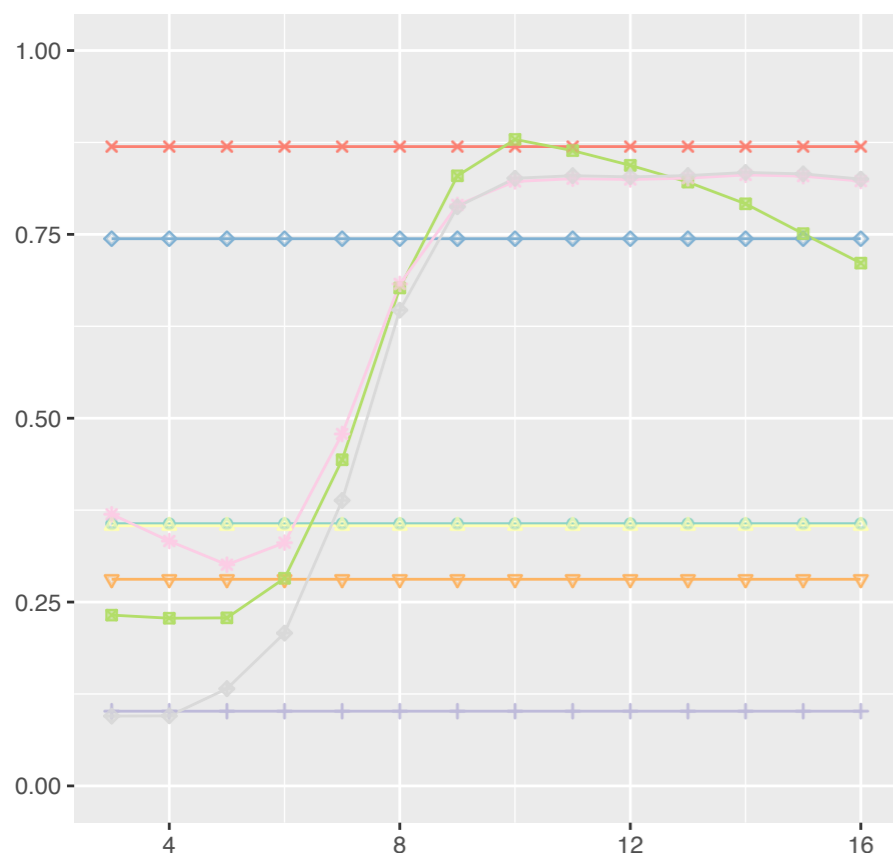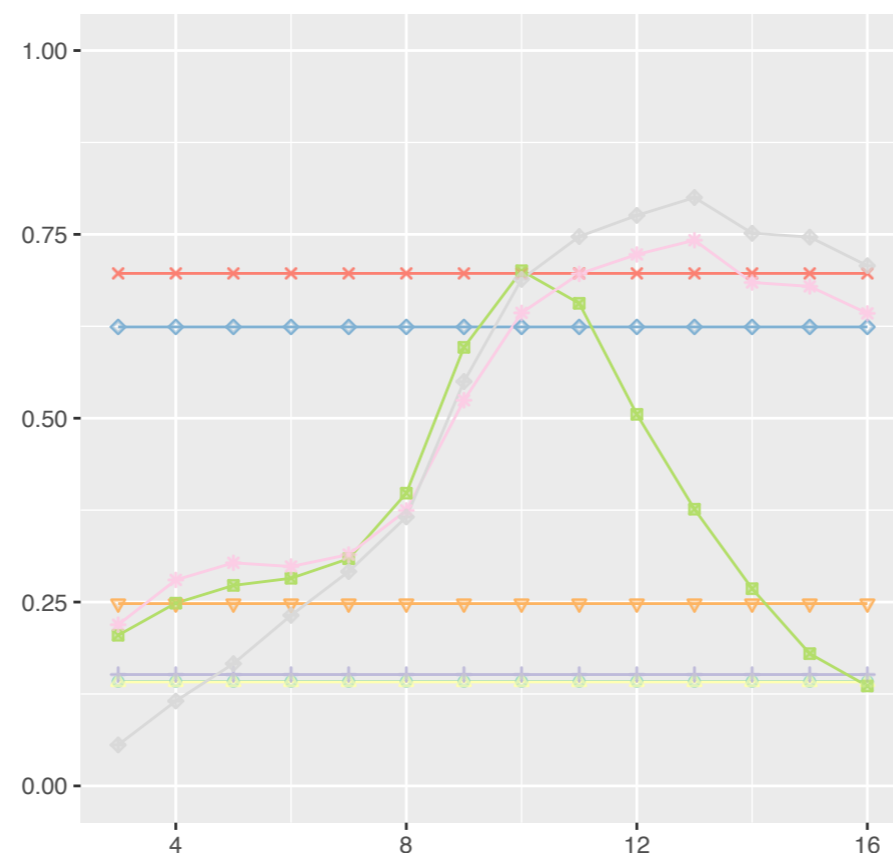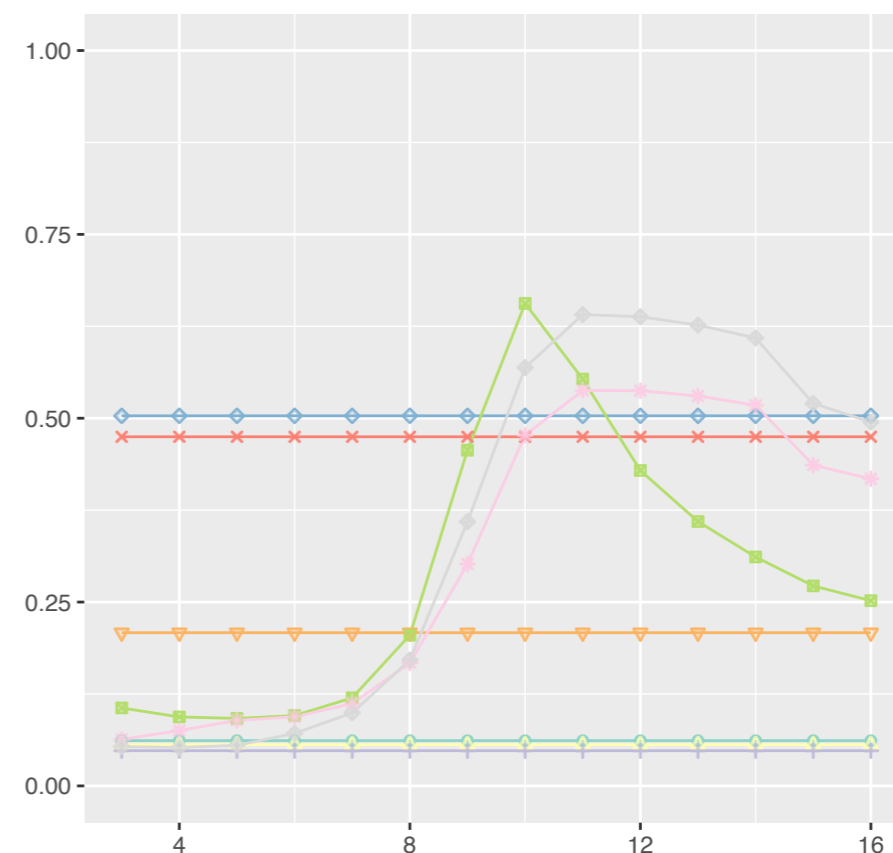

Transcript

n-gram

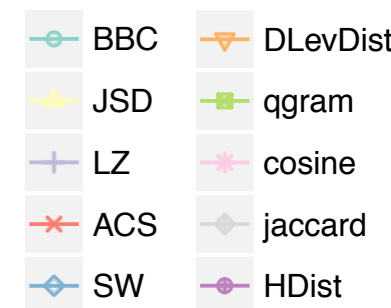

Supplement: Supplementary file 1 — Additional Figure 1. Protein-coding gene AUPR plots. Metric prediction performance computed on promoter and transcript sequences for annotate protein-coding homologs (AUPR on y-axis and n, the number of consecutive nucleotides in n-gram metrics, on x-axis). (PDF 158 kb) [file 12859_2018_2441_MOESM1_ESM.pdf]

Human-Mouse

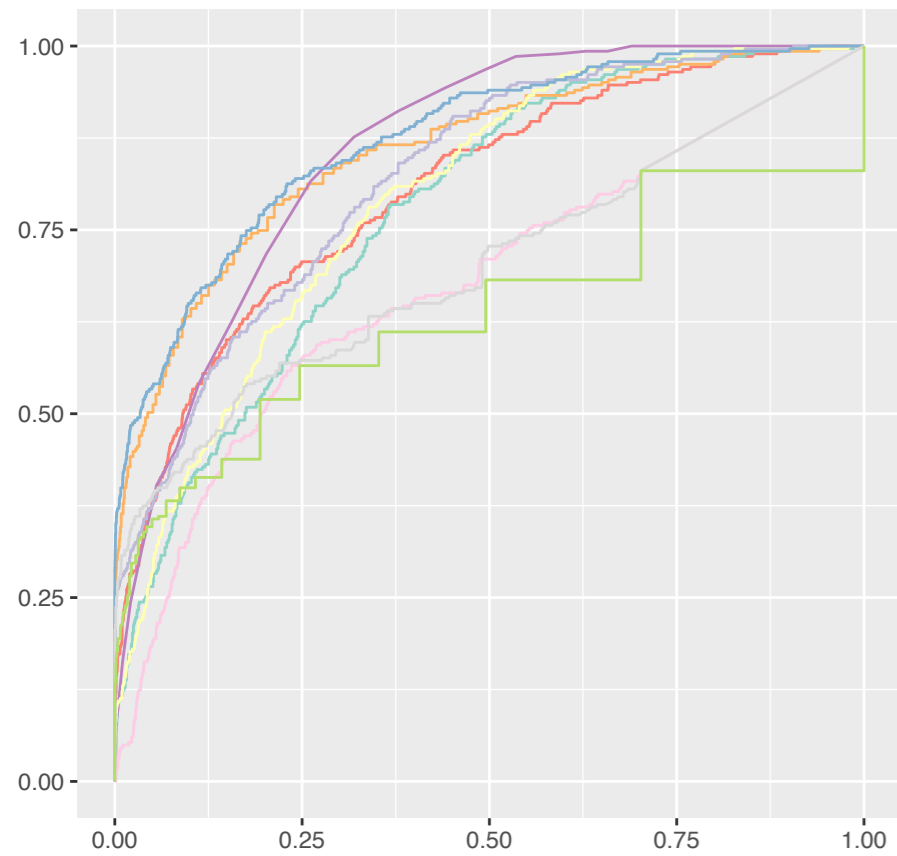

Mouse-Zebrafish

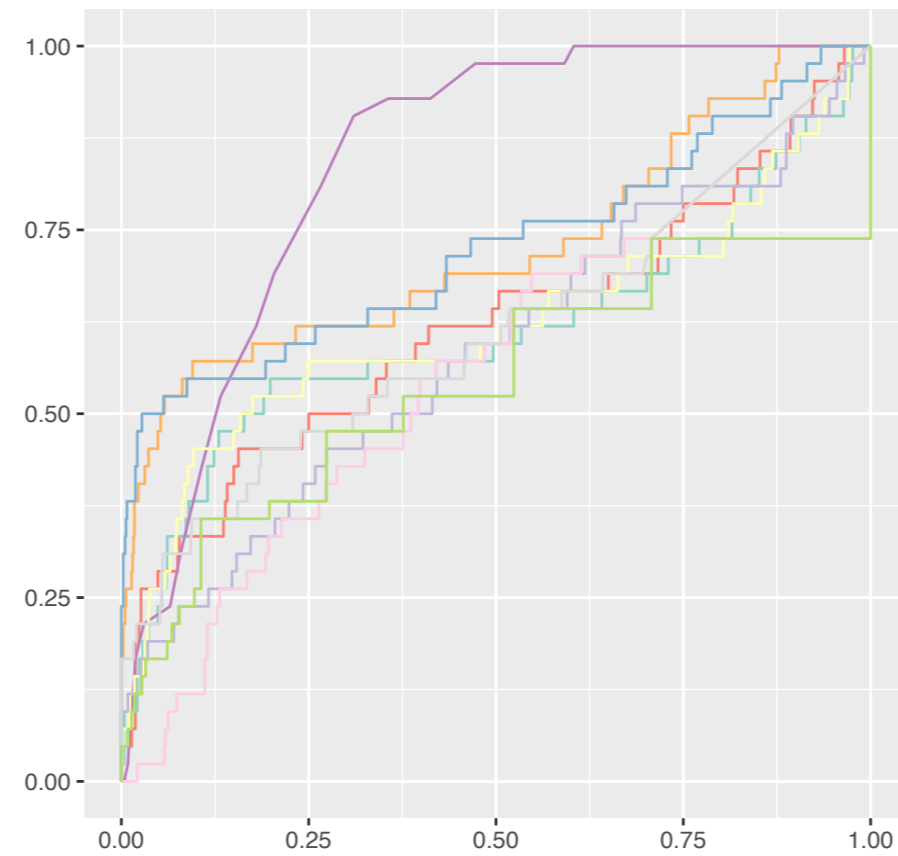

Human-Zebrafish

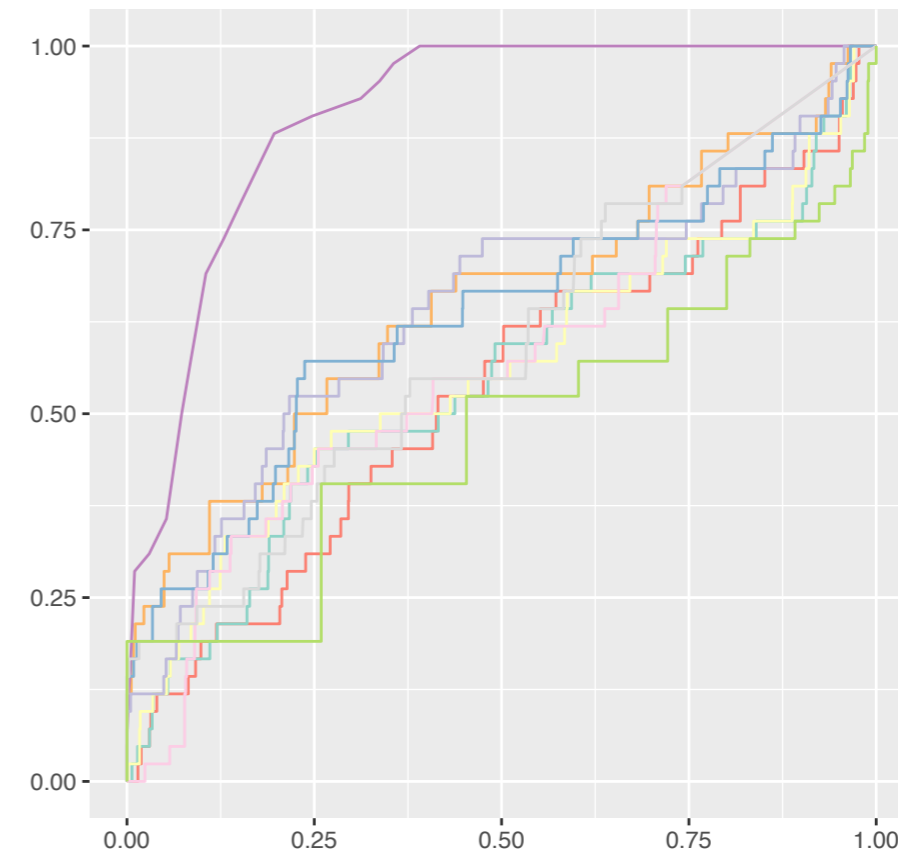

Promoter

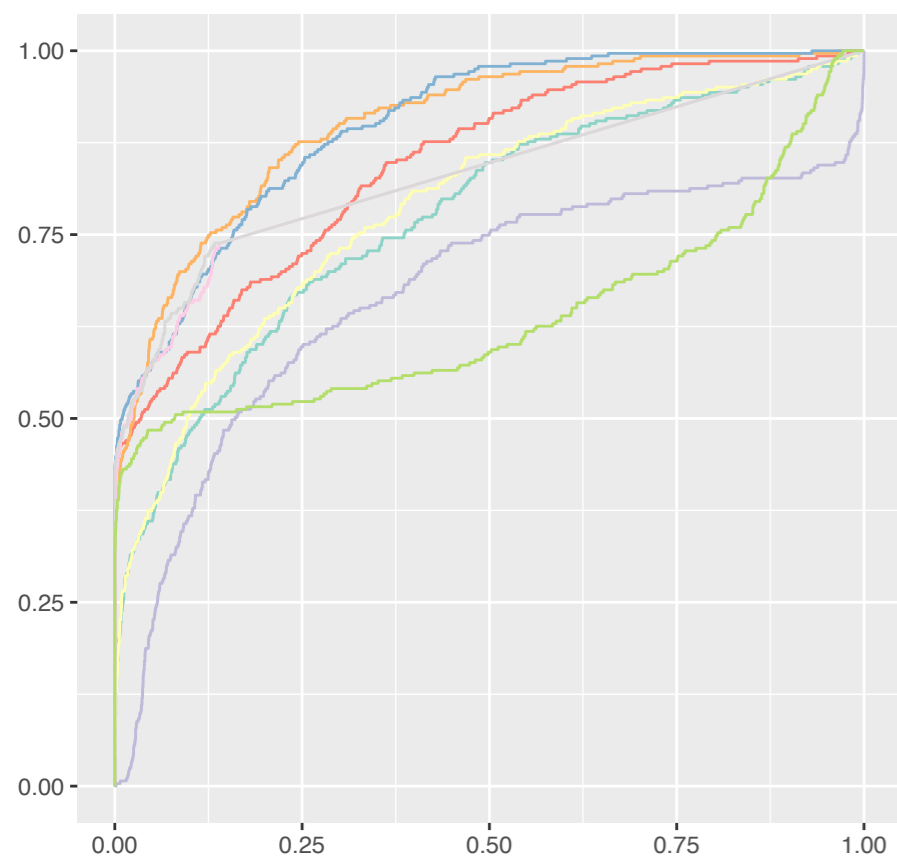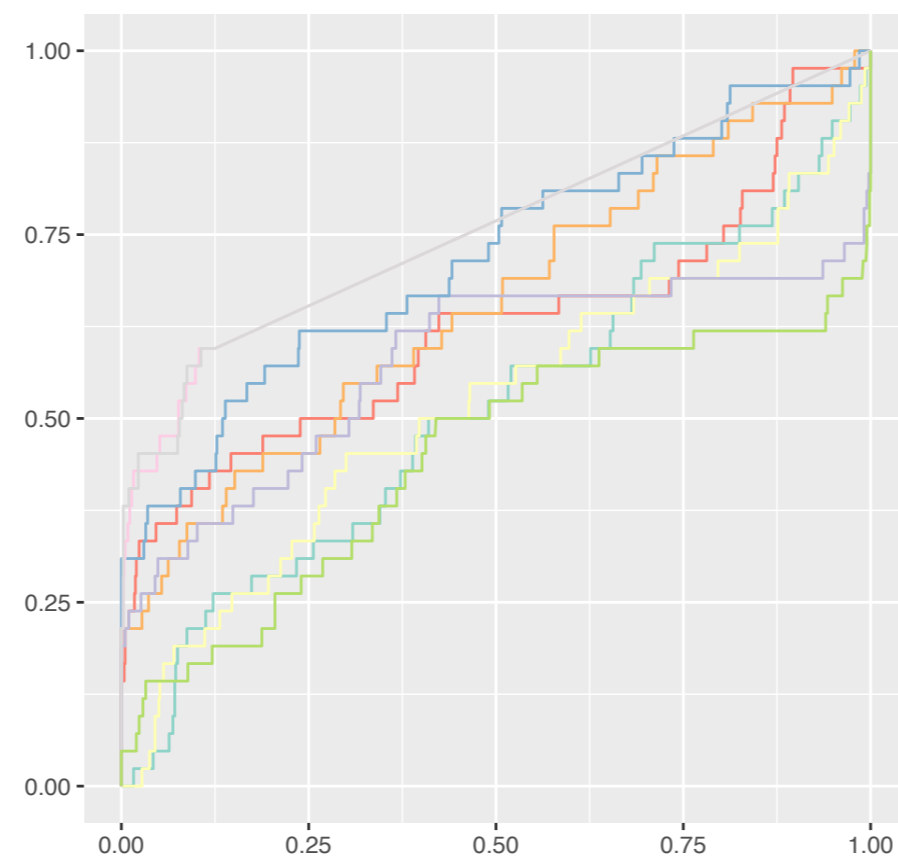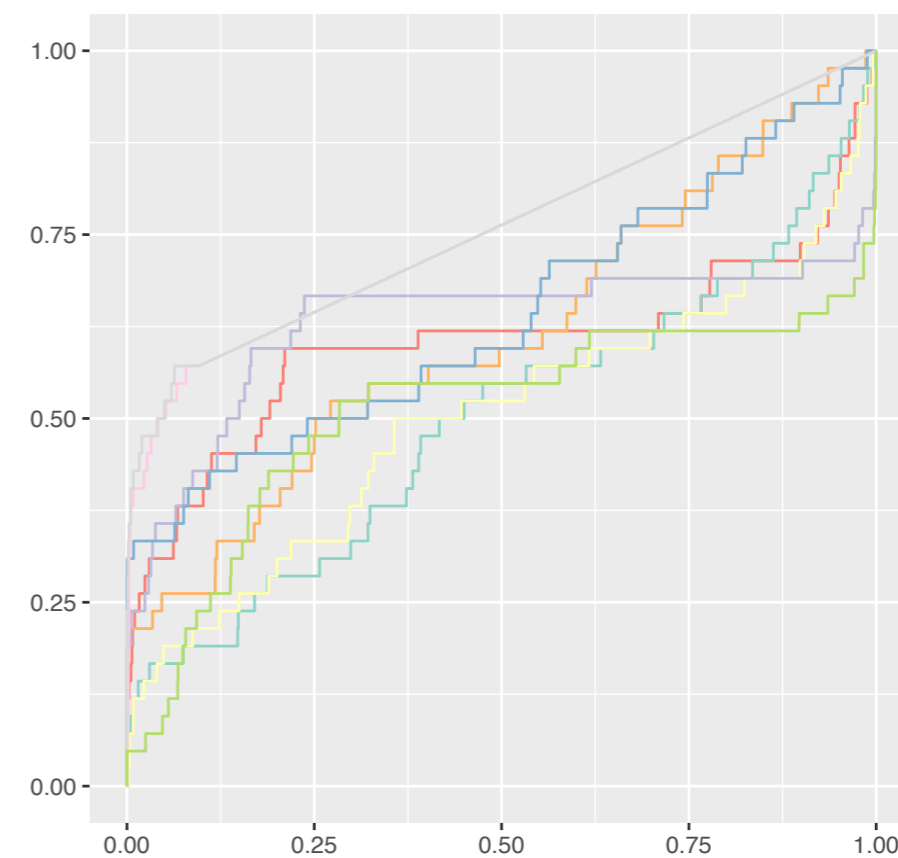

Transcript

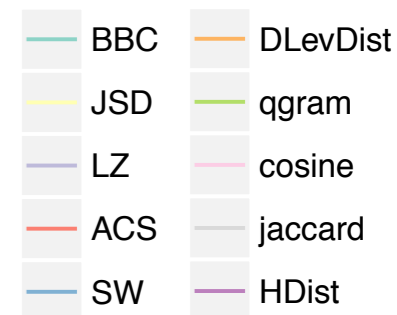

Supplement: Supplementary file 2 — Additional Figure 2. NONCODE ROC curves. ROC curves computed on promoter and transcript sequences for NONCODE lncRNA homologs (for n-gram metrics, n=12 has been chosen). (PDF 822 kb) [file 12859_2018_2441_MOESM2_ESM.pdf]

Human-Mouse

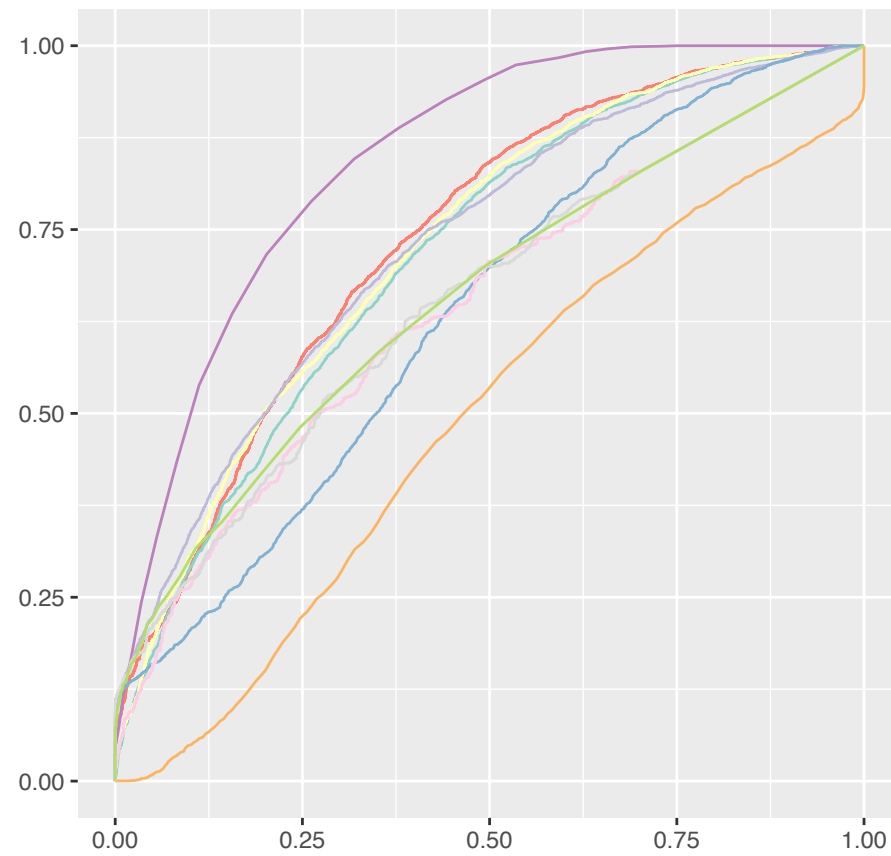

Mouse-Zebrafish

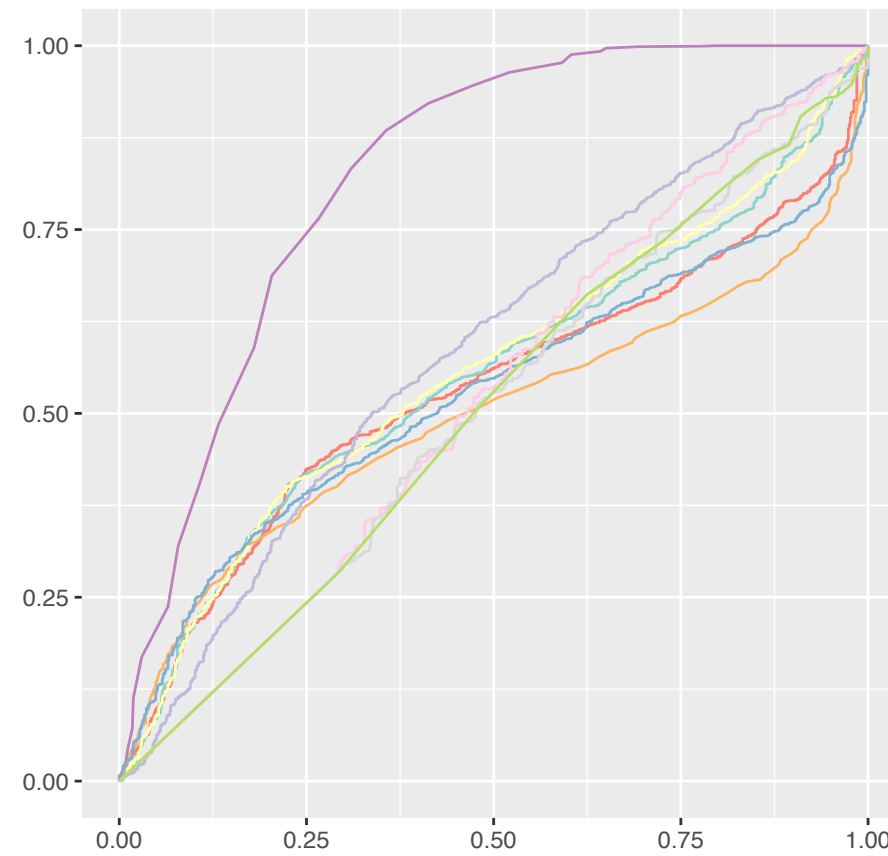

Human-Zebrafish

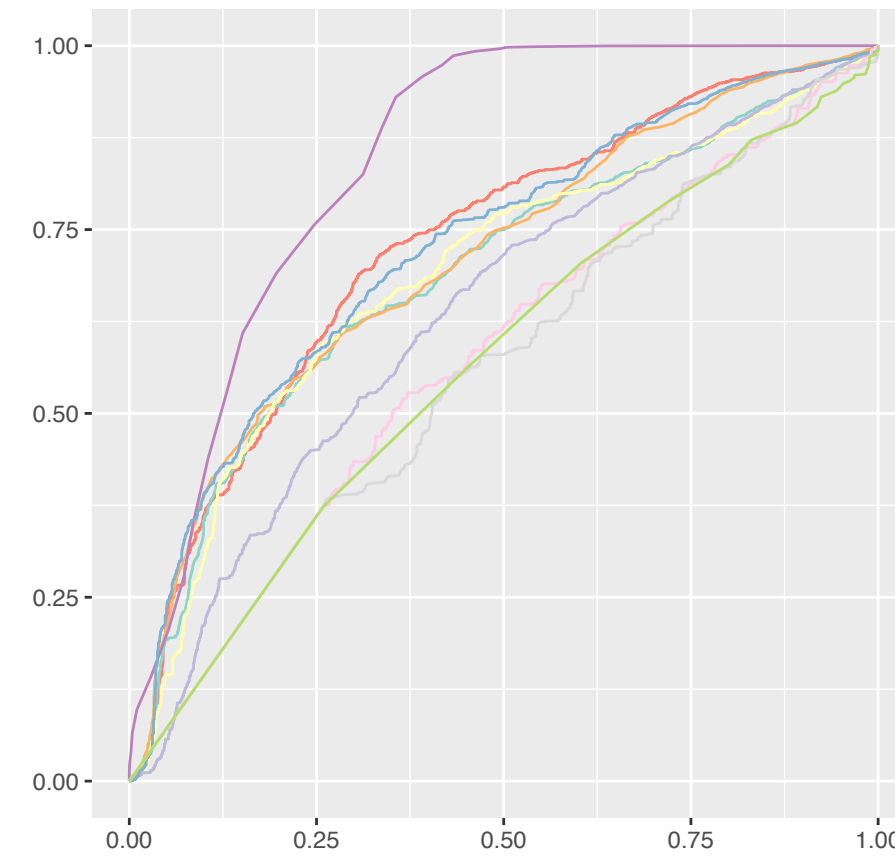

Promoter

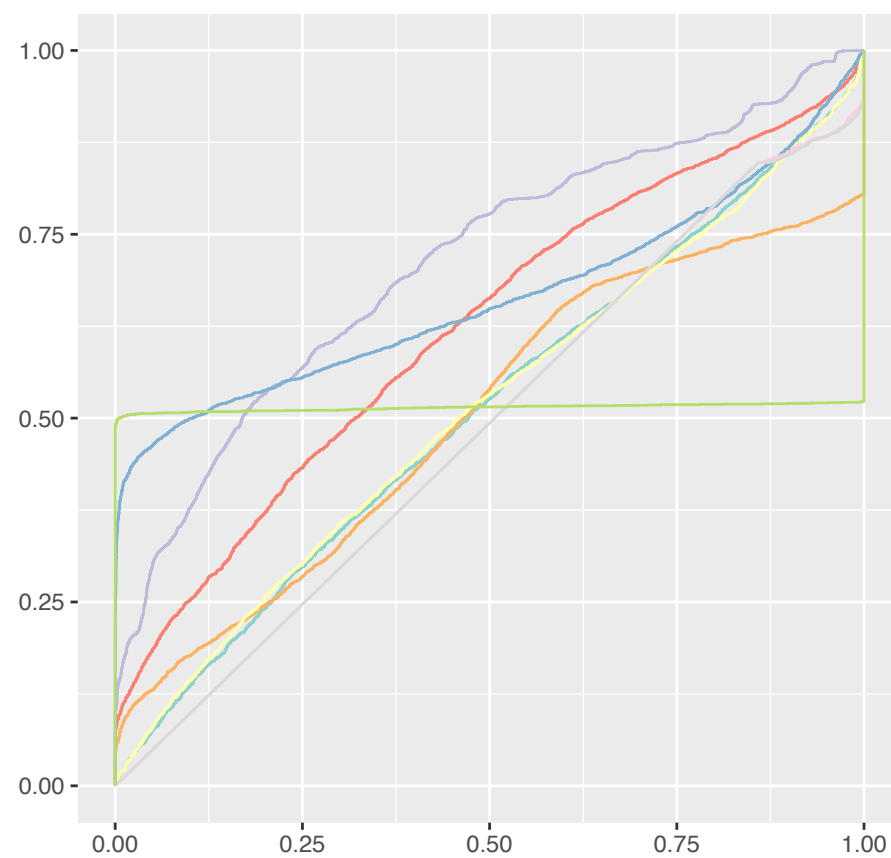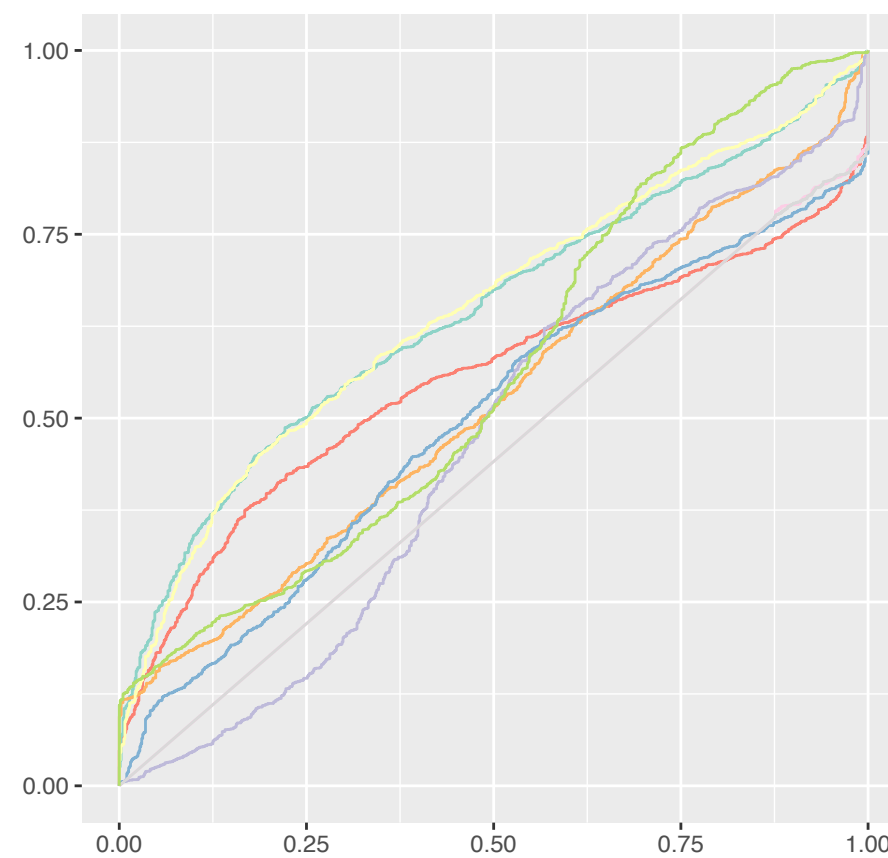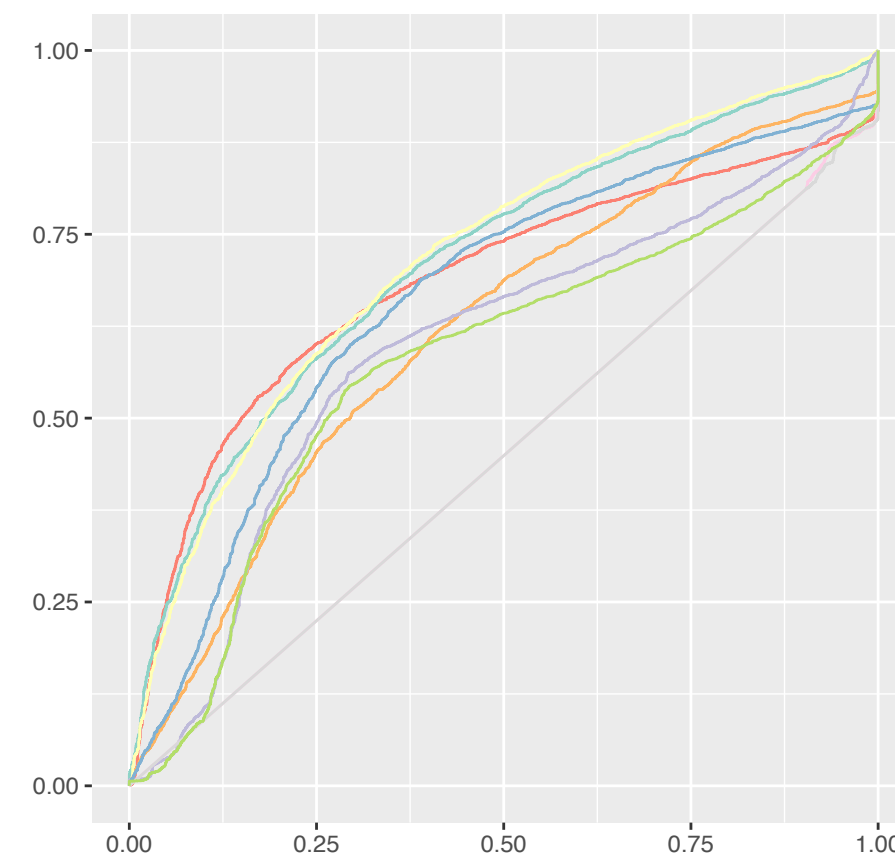

Transcript

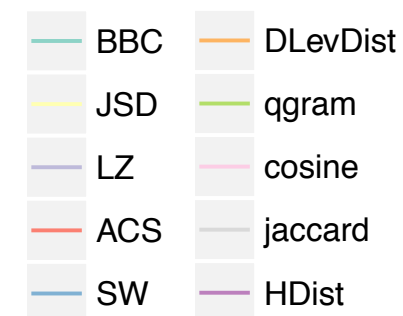

Supplement: Supplementary file 3 — Additional Figure 3. ZFLNC ROC curves. ROC curves computed on promoter and transcript sequences for ZFLNC lncRNA homologs (for n-gram metrics, n=12 has been chosen). (PDF 1580 kb) [file 12859_2018_2441_MOESM3_ESM.pdf]
